# Females Display Lower Risk of Myocardial Infarction From Higher Estimated Cardiorespiratory Fitness Than Males: The Tromsø Study 1994-2014

**DOI:** 10.1016/j.mayocpiqo.2023.12.007

**Published:** 2024-01-06

**Authors:** Edvard H. Sagelv, Andrea Casolo, Anne Elise Eggen, Kim Arne Heitmann, Kristoffer R. Johansen, Maja-Lisa Løchen, Ellisiv B. Mathiesen, Bente Morseth, Inger Njølstad, John O. Osborne, Karianne Hagerupsen, Sigurd Pedersen, Tom Wilsgaard

**Affiliations:** a School of Sport Sciences, Faculty of Health Sciences, UiT The Arctic University of Norway, Tromsø, Norway; b Department of Community Medicine, Faculty of Health Sciences, UiT The Arctic University of Norway, Tromsø, Norway; c Department of Clinical Medicine, Faculty of Health Sciences, UiT The Arctic University of Norway, Tromsø, Norway; d Department of Biomedical Sciences, University of Padua, Padua, Italy; e Department of Neurology, University Hospital of North Norway, Tromsø, Norway

## Abstract

**Objective:**

To examine the dose-response association between estimated cardiorespiratory fitness (eCRF) and risk of myocardial infarction (MI).

**Patients and Methods:**

Adults who attended Tromsø Study surveys 4-6 (Janurary 1,1994-December 20, 2008) with no previous cardiovascular disease were followed up through December 31, 2014 for incident MI. Associations were examined using restricted cubic splines Fine and Gray regressions, adjusted for education, smoking, alcohol, diet, sex, adiposity, physical activity, study survey, and age (timescale) in the total cohort and subsamples with hyperlipidemia (n=2956), hypertension (n=8290), obesity (n=5784), metabolic syndrome (n=1410), smokers (n=3823), and poor diet (n=3463) and in those who were physically inactive (n=6255).

**Results:**

Of 14,285 participants (mean age ± SD, 53.7±11.4 years), 979 (6.9%) experienced MI during follow-up (median, 7.2 years; 25th-75th, 5.3-14.6 years). Females with median eCRF (32 mL/kg/min) had 43% lower MI risk (subdistributed hazard ratio [SHR], 0.57; 95% CI, 0.48-0.68) than those at the 10th percentile (25 mL/kg/min) as reference. The lowest MI risk was observed at 47 mL/kg/min (SHR, 0.02; 95% CI, 0.01-0.11). Males had 26% lower MI risk at median eCRF (40 mL/kg/min; SHR, 0.74; 95% CI, 0.63-0.86) than those at the 10th percentile (32 mL/kg/min), and the lowest risk was 69% (SHR, 0.31; 95% CI, 0.14-0.71) at 60 mL/kg/min. The associations were similar in subsamples with cardiovascular disease risk factors.

**Conclusion:**

Higher eCRF associated with lower MI risk in females and males, but associations were more pronounced among females than those in males. This suggest eCRF as a vital estimate to implement in medical care to identify individuals at high risk of future MI, especially for females.

Cardiovascular diseases (CVD) are the leading causes of global mortality.^1^ One in 3 CVDs are coronary heart disease (CHD),^2^ of which myocardial infarction (MI) is the most severe CHD.^3^ Although MI incidence is decreasing in the Western world,^4^, ^5^, ^6^, ^7^ hospitalization rates due to MI have not declined in young individuals and are especially high among females, when compared with males.^7^, ^8^, ^9^ Young females also have higher in-hospital MI mortality than males.^8^ The American College of Cardiology and American Heart Association (ACC/AHA) guidelines on CVD prevention highlights missing data on risk assessment in young individuals.^10^ As such, early identification of these high-risk groups may further improve prevention of MI and aid in lowering the burden of CHD.^11^

Cardiorespiratory fitness (CRF) is consistently found to be an independent predictor of CHD^12^, ^13^, ^14^, ^15^, ^16^ and mortality,^13^^,^^17^ which has led to suggestions that CRF should be included as a vital measurement in routine medical care.^18^ However, the dose-response association between CRF and CHD appears equivocal between studies. Some studies report a J-shaped association,^12^^,^^13^^,^^15^^,^^16^^,^^19^, ^20^, ^21^ whereas others report an inverse, linear association, with the lowest CHD risks at the highest CRF level.^22^^,^^23^ These inconsistent observations may be attributed to categorization of CRF into study-specific centiles,^12^^,^^13^^,^^15^^,^^16^^,^^19^, ^20^, ^21^, ^22^, ^23^ which leads to loss of information^24^ and complicates translation to clinical decision making.^11^ Thus, using continuous CRF data may preserve information content and improve statistical power.^24^

Including cardiopulmonary exercise testing for direct CRF assessment in routine care is challenging owing to high costs, time, the need for specialized equipment, and skilled physiologists.^25^ However, estimated cardiorespiratory fitness (eCRF) from nonexercise prediction models is found to be reasonably accurate when compared with directly measured CRF,^26^ and eCRF is also associated with a lower risk of CHD^19^ and mortality.^20^, ^21^, ^22^ Therefore, eCRF may be a feasible option for routine assessment when direct cardiopulmonary testing is unavailable or unfeasible.^25^

Previous studies of CRF and CHD usually reported associations for all CHDs^12^^,^^13^^,^^20^, ^21^, ^22^ and not separate results for specific CHDs, such as MI, and many studies included only males.^13^, ^14^, ^15^ In studies examining CRF and MI risk including both sexes, results are inconsistent.^16^^,^^19^ In one study, higher CRF was associated with a lower MI risk in males but not in females,^16^ whereas in another study, only in females.^19^

In this study, we aimed to examine the dose-response association between eCRF in continuous data form and risk of MI in a large cohort of females and males and in subsamples of individuals with 1 or more CVD risk factors.

## Patients and Methods

### Study Sample and Design

This is a prospective cohort study with adult participants aged 25-86 years from the Tromsø Study, an ongoing population-based cohort study in Tromsø municipality, northern Norway.^27^ We included participants attending at least one of the Tromsø4-Tromsø6 surveys (Tromsø4 1994-1995, attendance: 77%; Tromsø5 2001, attendance: 79%; Tromsø6 2007-2008, attendance: 66%)^27^ because these include information on variables to estimate CRF (age, sex, waist circumference, self-reported physical activity, and resting heart rate). Additional inclusion criteria were information on education, alcohol, diet, and smoking. We excluded participants with present or previous CVD. If participants attended more than once, their earliest attendance was used. In total, 14,285 participants were included (Supplemental Figure 1, available online at http://www.mcpiqojournal.org), of which 7873 (55%) were females (Table).^28^

**Table:** Descriptive Characteristics of the Participants: The Tromsø Study 1994-2014

Characteristic	Total	Females	Males
Number of participants	14,285	7873	6412
Myocardial infarction	979 (6.9)	420 (5.3)	559 (8.7)
Incidence per 1000	7.6 (7.2-9.1)	5.7 (5.2-6.3)	10.2 (9.3-11.0)
Follow-up time (y)			
Median (25th-75th percentile)	7.2 (6.3-14.6)	9.5 (6.3-15.4)	7.0 (6.3-13.8)
Minimum-maximum	2.0-20.3	2.0-20.3	2.0-20.3
Age (y)	53.7±11.4	53.7±11.5	53.6±11.4
<30 y	589 (4.1)	320 (4.1)	269 (4.2)
30-39 y	1632 (11.4)	925 (11.8)	707 (11.0)
40-49 y	3277 (22.9)	1711 (21.7)	1566 (24.4)
50-59 y	4445 (31.1)	2573 (32.7)	1872 (29.2)
60-69 y	3550 (24.9)	1869 (23.7)	1681 (26.2)
≥70 y	792 (5.5)	475 (6.0)	317 (4.9)
Education			
Primary school	4608 (32.3)	2830 (36.0)	1778 (27.7)
High school	4504 (31.5)	2308 (29.3)	2196 (34.3)
University <4 y	2372 (16.6)	1134 (14.4)	11,238 (19.3)
University ≥4 y	2801 (19.6)	1601 (20.3)	1200 (18.7)
Adiposity markers			
Body mass index (kg/m^2^)	26.3±4.2	26.0±4.5	26.6±3.6
<25	5953 (41.7)	3714 (47.2)	2239 (34.9)
25-29	6044 (42.3)	2849 (36.2)	3195 (49.9)
≥30	2281 (16.0)	1307 (16.6)	974 (15.2)
Central obesity, n (%)^a^	5784 (32.5)	3300 (38.5)	2484 (25.2)
Diet quality (nutritional guideline)	1.7±0.7	1.7±0.7	1.7±0.7
<1 nutritional guideline	3463 (24.3)	1916 (24.3)	1547 (24.1)
1-2 nutritional guideline	7347 (51.4)	4034 (51.2)	3313 (51.7)
≥2 nutritional guideline	3475 (24.3)	1923 (24.4)	1552 (24.2)
Alcohol intake	2.5±3.2	1.8±2.4	3.3±3.8
Teetotaler	2974 (20.8)	2050 (26.0)	924 (14.4)
0.1-1.9 units/wk	6139 (43.0)	3747 (47.6)	2392 (37.3)
2.0-3.9 units/wk	2880 (20.2)	1326 (16.8)	1554 (24.2)
≥4.0 units/wk	2292 (16.0)	750 (9.5)	1542 (24.1)
Smoking			
Current smoker	3823 (26.8)	2122 (27.0)	1701 (26.5)
Previous smoker	5219 (36.5)	2554 (32.4)	2665 (41.6)
Never smoker	5243 (36.7)	3197 (40.6)	2046 (31.9)
Physical activity (MET-h/wk)	9.4±7.5	9.1±7.1	9.7±7.9
<7.5 MET-h/wk	6255 (43.8)	3464 (44.0)	2791 (43.5)
<7.5-15 MET-h/wk	5603 (39.2)	3196 (40.6)	2407 (37.5)
≥15 MET-h/wk	2427 (17.0)	1213 (15.4)	1214 (18.9)
eCRF (mL/kg/min)	36.3±8.0	32.3±6.2	41.1±7.4
Disease	632 (4.4)	361 (4.6)	271 (4.2)
Cancer	365 (2.6)	247 (3.1)	118 (1.8)
Diabetes	397 (2.8)	207 (2.6)	190 (3.0)
Cardiometabolic health			
Hypertension	8290 (58.0)	4093 (52.0)	4197 (65.5)
Hyperlipidemia	2956 (20.7)	1838 (23.4)	1118 (17.5)
Metabolic syndrome	1310 (9.2)	845 (10.8)	465 (7.3)

eCRF, estimated cardiorespiratory fitness; MET, metabolic equivalent of task.

Data are shown as frequency (%), mean ± SD, or rate (95% CI).

a Central obesity is defined as waist circumference (cm) thresholds at specific body mass index thresholds, as described by Ross et al.^28^.

### Ethical Considerations

The Tromsø Study surveys were conducted according to the Declaration of Helsinki. All participants provided written informed consent. The Regional Ethics Committee for Medical and Health Research Region North approved this study (Ref.: 2016/1792).

### Diagnosis of MI

Incident MI diagnosis was identified through linkage to the diagnosis registry at the University Hospital of North Norway, the only hospital serving Tromsø municipality,^29^ and the Norwegian Cause of Death Registry,^30^ searching for International Classification of Disease (ICD), ninth edition, codes 410-414, 427, 428, 798, and 799 and ICD-10 codes I20-I25, I46-I48, I50, R96, R98, and R99. In addition, manual and/or electronic text searches for notes on MI were completed with paper (used until 2001) and digital hospital records for all participants with a diagnosis of ICD-8 and ICD-9 codes 430-438 and ICD-10 codes I60-I69, G45, G46, or G81.^29^ Experienced physicians reviewed and validated all diagnoses on the basis of hospital records and, when available, death certificates and autopsy reports. Review of medical records minimizes misclassification in data collection from health registries.^29^ Emigration and moving date were retrieved from the Norwegian Population Registry. Participants were followed up from baseline to incident MI, migration, moving from Tromsø, death, or end of follow-up on December 31, 2014, whichever came first. To exclude participants with present or previous CVD, we used MI records before baseline participation and self-reported present or a history of CVD (ie, MI, angina pectoris, and stroke).

### Estimated CRF

Two nonexercise prediction formulas were used to estimate CRF from self-reported physical activity, waist circumference, age, sex, and resting heart rate (Supplemental File 1, available online at http://www.mcpiqojournal.org). For participants in Tromsø4-5 (1994-1995 and 2001, respectively), we used the formula by Nauman et al,^21^ being based on the Cohort of Norway physical activity questionnaire^31^ that was used in Tromsø4-5 (Supplemental Table 1, available online at http://www.mcpiqojournal.org). For participants in Tromsø6 (2007-2008), we used the formula by Nes et al,^32^ which is based on the physical activity frequency, intensity, and duration questionnaire that was used in Tromsø6 (Supplemental Table 2, available online at http://www.mcpiqojournal.org). Different algorithms were fitted because of different physical activity questionnaires in Tromsø4-5 vs Tromsø6. Nevertheless, both formulas are validated in the same cohort sample and should, thus, represent the same CRF values, which both explain ∼60% of the variance in directly measured CRF from a test to exhaustion using indirect calorimetry.^21^^,^^32^ From these formulas, we expressed eCRF as maximal oxygen uptake in milliliter per kilogram body weight per minute (mL/kg/min) (Supplemental File 1).

### Covariates

On the basis a directed acyclic graph, we identified education, smoking, alcohol intake, diet, age, sex, adiposity, and physical activity as potential confounding sources in the association between eCRF and MI (Supplemental Figure 1). Because age, sex, waist circumference, and physical activity are included in eCRF formula, education, smoking, alcohol intake, and diet were included as potential confounders. Educational level was categorized into primary school, high school, university <4 years, and university ≥4 years (Supplemental File 2, available online at http://www.mcpiqojournal.org). Smoking was categorized as current, previous, or never. We harmonized alcohol intake (units/wk) from multiple questions on alcohol intake (Supplemental File 3 and Supplemental Tables 3-5, available online at http://www.mcpiqojournal.org). Diet quality was harmonized according to national nutritional guidelines^33^ on a scale from 0.0 to 4.0 of fruit, saturated fat, fish, and processed meat intake from multiple questions on food intake (Supplemental File 4 and Supplemental Tables 6-8, available online at http://www.mcpiqojournal.org).

### Definition of Subsamples With Additional CVD Risk Factors

Hypertension (yes/no) was defined by a combination of questionnaires, reported medicine use (Anatomical Therapeutic Chemical: C02, C03, C07, C08, and C09), and blood pressure recordings (Supplemental File 5, available online at http://www.mcpiqojournal.org). Hyperlipidemia (yes/no) was defined by serum total cholesterol (≥5.17 mmol/L), questionnaires, and reported medicine use (Anatomical Therapeutic Chemical: C10) (Supplemental File 5). Central obesity was defined as specific waist circumference (in centimeters) thresholds at specific body mass index (calculated as the weight in kilograms divided by the height in meters squared) categories (normal weight, overweight, obese, and obese class II) according to Ross et al^28^ (Supplemental File 5). Metabolic syndrome was defined according to the International Federation of Diabetes^34^ (Supplemental File 5).

Physical inactivity was defined as reporting <7.5 metabolic equivalents of tasks (METs) per week of moderate intensity (equivalent to the lower-limit physical activity guideline^35^), which we calculated from the Cohort of Norway physical activity questionnaire (Tromsø4-5, 1994-1995 and 2001, respectively) (Supplemental Table 7) and the physical activity frequency, intensity, and duration questionnaire (Tromsø6, 2007-2008) (Supplemental Table 8).

### Statistical Analyses

To examine the dose-response association between eCRF and MI, we used restricted cubic splines in Fine and Gray regressions^36^ to account for competing risks of death from other causes than MI. We performed analyses separately by sex because CRF^21^ and MI risk^9^ differ by sex. We further examined the associations in subsamples with hypertension, hyperlipidemia, metabolic syndrome, central obesity, being physically inactive, or not meeting any nutritional guidelines and in those having ≥2, ≥3 and ≥4 CVD risk factors. As metabolic syndrome is a composite of multiple CVD risk factors,^34^ it was not included in the summation of ≥2, ≥3 and ≥4 CVD risk factors. We adjusted all analyses for education, smoking, diet quality, alcohol intake, study survey (dummy variable), and age as timescale.^37^ Waist circumference and physical activity were included in the eCRF formulas^21^^,^^31^ and consequently not additionally adjusted for to avoid multicollinearity. Participants entered the analyses 2 years after study attendance (left truncation).

Using time-dependent weights as described by Lambert,^38^ we calculated modified weighted Schoenfeld residuals to test proportional subdistributed hazards by goodness-of-fit tests by Zhou et al^39^; all covariates indicated proportional subdistributed hazards (all *P*>.12) except smoking in both models (females: *P*=.004; males: *P*=.01). However, the log-log survival plot of subdistributed hazards displayed reasonable parallel lines between subgroups of smoking status (Supplemental Figures 2 and 3, available online at http://www.mcpiqojournal.org). Knots in the restricted cubic splines were placed at the 10th, 50th, and 90th percentiles of the distribution of eCRF. The reference value for dose-response splines were set at the 10th percentile of the distribution, separately by sex (males: 32 mL/kg/min; females: 25 mL/kg/min). Changing knot placements or knot numbers did not change interpretation of the spline slopes. Wald tests indicated departure from linearity in all models (all *P*<.004).

For sensitivity analyses, we performed the following: (1) examined the associations in those aged <60 years and >60 years to evaluate whether age had large influence on the association magnitude (age is inversely associated with CRF^31^^,^^40^^,^^41^); (2) created age-specific quintiles of eCRF in accordance with recommendations to limit the influence of age in CRF-health outcome associations^40^; and (3) set study entry 5 years after study attendance to evaluate the influence of reverse causation bias. All analyses were performed using Stata version 17 (StataCorp) with an α at 0.05. Data are shown as subdistributed hazard ratio (SHR) with 95% CIs and as frequency (%) or mean ± SD for descriptive data.

## Results

Of the total 14,285 participants, 979 (6.9%) experienced an MI during the median 7.2 follow-up years (interquartile range, 8.3 years) (Table). Survey-specific descriptive characteristics are found in Supplemental Table 9 (available online at http://www.mcpiqojournal.org).

**Figure 1:**
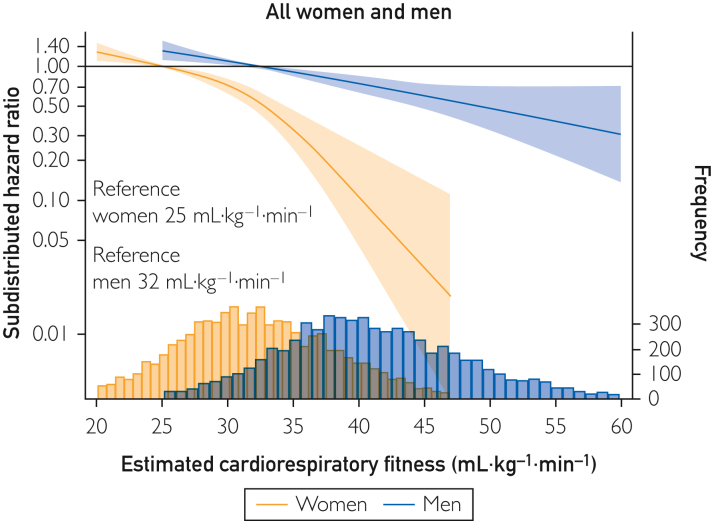
Restricted cubic spline Fine and Grey regressions of eCRF and risk of MI among females and males: the Tromsø Study 1994-2014. Data are shown as subdistributed hazard ratio (line) with 95% CIs (shaded area), adjusted for education, smoking, diet, alcohol, and study survey; age, waist circumference (ie, adiposity), and physical activity are adjusted by inclusion in the eCRF formulas. Reference of the spline is set to the 10th percentile of the distribution of the group (ie, among females and males separately), and values are shown between the first and 99th percentile of the distribution of estimated cardiorespiratory fitness. Frequency refers to the frequency of observed eCRF. eCRF, estimated cardiorespiratory fitness; MI, myocardial infarction.

Higher eCRF was associated with a lower risk of MI in both females and males in an exponential pattern, which was more pronounced for females than in males (Figure 1). Compared with 25 mL/kg/min (10th percentile), females having an eCRF corresponding to median (32 mL/kg/min) displayed a 43% lower MI risk (SHR, 0.57; 95% CI, 0.48-0.68), and maximal risk reduction was observed at 47 mL/kg/min (SHR, 0.02; 95% CI, 0.01-0.11) (Figure 1). Males with median eCRF (40 mL/kg/min) displayed a 26% lower MI risk (SHR, 0.74; 95% CI, 0.63-0.86) than those with reference 32 mL/kg/min (10th percentile) (Figure 1). Maximal risk reduction for males was observed at 60 mL/kg/min (SHR, 0.31; 95% CI, 0.14-0.71) (Figure 1).

**Figure 2:**
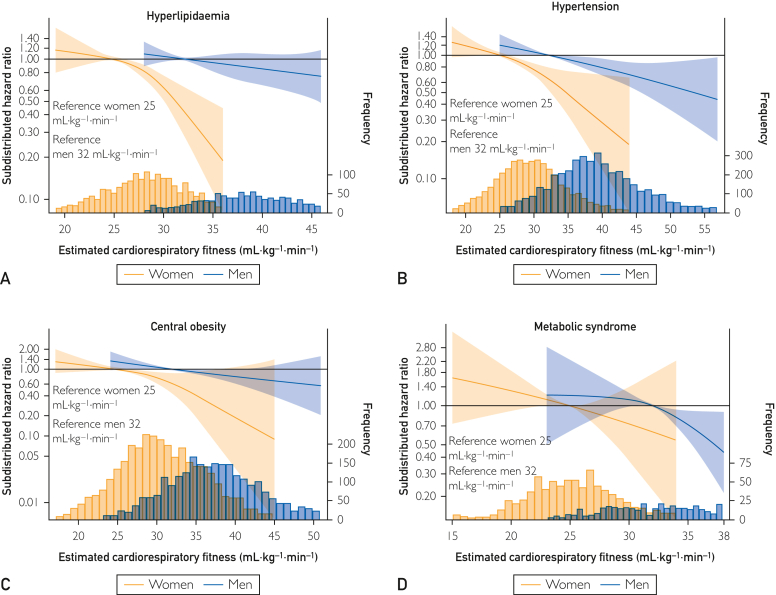
Restricted cubic spline Fine and Grey regressions of eCRF and risk of MI among females and males with (A) hyperlipidemia (n=2965, MI=400); (B) hypertension (n=8290, MI=834); (C) central obesity (n=5784, MI=379); and (D) metabolic syndrome (n=1410, MI=181): the Tromsø Study 1994-2014. Data are shown as subdistributed hazard ratio (line) with 95% CIs (shaded area), adjusted for education, smoking, diet, alcohol, and study survey; age, waist circumference (ie, adiposity), and physical activity are adjusted by inclusion in the eCRF formulas. Reference of the spline is set to the 10th percentile of the distribution of the group (ie, among females and males separately), and values are shown between the first and 99th percentile of the distribution of estimated cardiorespiratory fitness. Frequency refers to the frequency of observed eCRF. eCRF, estimated cardiorespiratory fitness; MI, myocardial infarction.

**Figure 3:**
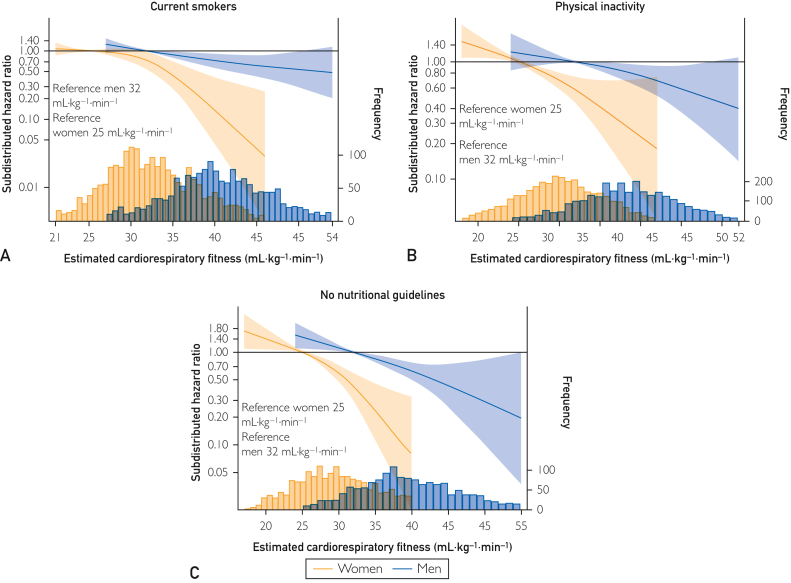
Restricted cubic spline Fine and Grey regressions of eCRF and risk of MI among females and males who (A) are smokers (n=3823, MI=372); (B) are physically inactive (n=6255, MI=443); and (C) do not meet any nutritional guideline (n=3463, MI=382): the Tromsø Study 1994-2014. Data are shown as subdistributed hazard ratio (line) with 95% CIs (shaded area), adjusted for education, smoking, diet, alcohol, and study survey; age, waist circumference (ie, adiposity), and physical activity are adjusted by inclusion in the eCRF formulas. Reference of the spline is set to the 10th percentile of the distribution of the group (ie, among females and males separately), and values are shown between the first and 99th percentile of the distribution of estimated cardiorespiratory fitness. Frequency refers to the frequency of observed eCRF. eCRF, estimated cardiorespiratory fitness; MI, myocardial infarction.

**Figure 4:**
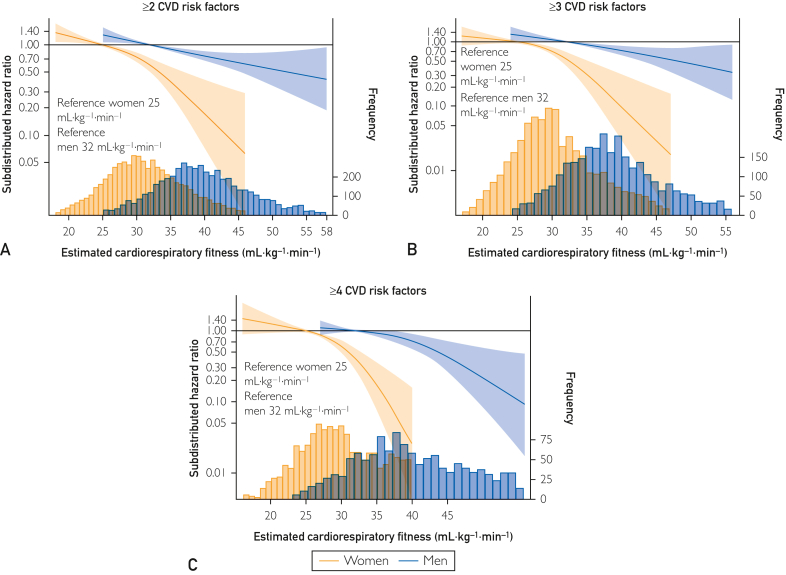
Restricted cubic spline Fine and Grey regressions of eCRF and risk of MI among females and males with (A) 2 or more CVD risk factors (n=9720, MI=857); (B) 3 or more CVD risk factors (n=5359, MI=600; and (C) 4 or more CVD risk factors (n=1982, MI=293): the Tromsø Study 1994-2014. The CVD risk factors included are smoking, hypertension, hyperlipidemia, obesity, poor diet, and physical inactivity. Data are shown as subdistributed hazard ratio (line) with 95% CIs (shaded area), adjusted for education, smoking, diet, alcohol, and study survey; age, waist circumference (ie, adiposity), and physical activity are adjusted by inclusion in the eCRF formulas. Reference of the spline is set to the 10th percentile of the distribution of the group (ie, among females and males separately), and values are shown between the first and 99th percentile of the distribution of eCRF. Frequency refers to the frequency of observed eCRF. CVD, cardiovascular disease; eCRF, estimated cardiorespiratory fitness; MI, myocardial infarction.

Compared with the total sample (Figure 1), the association magnitudes and spline slopes were similar in subsamples with 1 other CVD risk factor (Figures 2 and 3), and even with 4 or more CVD risk factors, higher eCRF was associated with a lower risk of MI (Figure 4).

### Sensitivity Analyses

When splitting the analyses to those aged <60 years and ≥60 years, the association patterns were similar but slightly attenuated for those aged >60 years; 40 mL/kg/min in eCRF was associated with a 81% lower MI risk in females (SHR, 0.17; 95% CI, 0.07-0.40) and 23% lower MI risk in males (SHR, 0.75; 95% CI, 0.61-0.93) compared with the reference 10th percentile (25 and 32 mL/kg/min, respectively) (Supplemental Table 10, available online at http://www.mcpiqojournal.org). When using age-specific quintiles of eCRF, higher eCRF quintiles were associated with lower MI risks in both females and males, but the point SHR estimate was greater in females (quintile 1 vs 5; SHR, 0.58; 95% CI, 0.41-0.83) than that in males (quintile 1 vs 5; SHR, 0.71; 95% CI, 0.54-0.93) (Supplemental Tables 11 and 12, available online at http://www.mcpiqojournal.org). The results remained unchanged when restricting study entry to 5 years after survey attendance (Supplemental Table 13, available online at http://www.mcpiqojournal.org).

## Discussion

In this prospective cohort study, higher eCRF was associated with a substantial lower risk of MI in an exponential pattern in both females and males. However, the lower risk was more pronounced in females than that in males. These patterns of association were also evident among those with 1 or more CVD risk factors and in sensitivity analyses of those older than 60 years.

Previous studies including both fatal and nonfatal MI and including both sexes have reported conflicting results regarding the association between CRF and MI separately for females and males, with 1 study reporting an association only in females,^19^ whereas another only in males.^16^ In this study, higher eCRF was associated with a lower MI risk in both females and males. However, using continuous eCRF data in restricted cubic splines, this study illustrated that the dose-response association between higher eCRF and MI was nonlinear and more pronounced in females than that in males. For example, an eCRF corresponding to 32 mL/kg/min in maximal oxygen uptake (equivalent to 9.1 METs in a maximal exercise test) for females and 45 mL/kg/min (equivalent to 12.9 METs in a maximal exercise test) for males would indicate a ∼40% lower risk of MI; higher eCRF levels would indicate an even lower MI risk.

Although MI incidence and mortality is decreasing in the Western world,^2^^,^^4^, ^5^, ^6^, ^7^ preventive measures can further decrease CHD mortality and morbidity.^11^ The ACC/AHA guidelines on CVD prevention highlights the importance of identifying those who will benefit most from preventive measures, especially as risk assessment for young individuals are lacking.^10^ Acute MI more often manifests silently in females than that in males,^9^ and more young females than males are hospitalized owing to MI,^8^^,^^9^ leading to a greater comorbidity burden among females than that in males.^9^ The dose-response curves for CRF as observed in this study may aid clinicians in evaluating risks of future MI among their patients separately for females and males, with simple use of self-reported physical activity, waist circumference, and resting heart rate. Hence, our findings indicate that eCRF may aid as an essential early MI risk identifier, especially among females.

Moreover, although previous studies also have observed an association between CRF and CHD in individuals with established CVD risk factors, such as hypertension,^42^ hyperlipidemia,^43^ smoking,^43^ and obesity,^44^ this is the first study using eCRF to examine the association with fatal and nonfatal CHD among females and males with CVD risk factors. Thus, even among those with other established risk factors for CVD, eCRF may be able to identify those at even higher risk of MI. Indeed, previous studies have indicated that including directly measured CRF^12^ or eCRF^21^ improves the risk prediction of CHD beyond traditional risk factors.

The lower MI risk with higher eCRF in females compared with that in males may be because females on average experience MI at older age than males^9^; because age explains most of the variance (∼30%) in the eCRF formulas,^21^^,^^31^ this could inflate the association magnitude. However, the sensitivity analysis including only individuals older than 60 years displayed a substantially lower MI risk in females, indicating that females may derive greater MI risk reduction from a high CRF than males,^19^ as also observed for physical activity and CHD.^45^ Alternatively, males overreport their physical activity level to a greater extent than females^46^; because the eCRF formulas include self-reported physical activity,^21^^,^^31^ the sex difference in the association magnitude may also partly be explained by greater regression dilution bias in results for males than that for females.

### Strengths

In this study, our use of restricted cubic splines illustrates that eCRF is nonlinearly and exponentially associated with a lower risk of MI in both females and males. These substantial magnitudes are likely attributed to our use of continuous eCRF data that preserve information quality and statistical power,^24^ which is easier to transfer to clinical decision making than arbitrary study-specific categorized data.^11^ Moreover, we used Fine and Gray regressions to account for competing risk of other causes of death.^36^ It is previously shown that traditional time-to-event analysis may overestimate the risk of CVD because it handles death as censoring instead of a competing risk for the outcome of interest.^47^

Finally, we used carefully validated health registry data,^28^ limiting the risk of misclassification and identifying both fatal and nonfatal MI events. This, combined with the population-based cohort study design with a high attendance, strengthens generalizability of our findings toward Western populations. Moreover, with such data, the exposure estimate (ie, eCRF), and thus the outcome effect (ie, MI risk), is likely more accurate than if using solely registry data because the covariate distribution in the sample is likely to be representative of the base population.^11^

### Limitations

Although our study is likely representative of Western populations, CVD mortality and morbidity is greatest in low-income and middle-income countries.^1^, ^2^, ^3^^,^^48^ However, because higher physical activity levels associate with lower risk of CVD mortality in all world regions,^49^ and physical activity improves CRF,^50^ influence of CRF is likely of similar biological effect, even in different regions or ethnicities. For example, higher CRF is found to be inversely associated with MI risk in Trinidadian males.^51^ Nevertheless, research using eCRF in low-income and middle-income countries is still warranted to confirm and test the implementation and feasibility of eCRF in these regions.

Higher age is inversely associated with CRF.^52^ Because age is also the greatest component of the eCRF formulas used,^31^ this may limit the possibility to examine an effect of CRF independent of age.^40^ When examining age-specific eCRF quintiles, the association magnitudes were attenuated at the highest ends of eCRF but mostly revealed similar findings as the spline modeling (ie, our main analyses). However, grouping continuous data leads to loss of information and statistical power.^24^ Nevertheless, it is well known and should be acknowledged that CRF declines with increasing age.^53^ Thus, it is still likely that increasing physical activity levels^50^ or engaging in structured exercise^54^ of sufficient volume and intensity will maintain or improve CRF at all ages, which potentially can aid in preventing MI and lower CHD burden and mortality.

Physical activity was self-reported, which is influenced by information bias.^46^ Thus, we likely misclassified some individuals as active and inactive when performing separate analysis of those meeting and not meeting current physical activity guidelines.^55^ This may also influence eCRF as mentioned earlier. Indeed, the CRF formulas are less precise for outliers (the most-fit and least-fit individuals) when compared against an exercise test to exhaustion using indirect calorimetry.^31^ This is likely a result of a generally nonlinear association between eCRF and directly measured CRF.^56^ Nevertheless, despite some misclassification, the simplicity and reported utility^26^ of eCRF make a case for including this measure in routine medical care.^25^

## Conclusion

In this prospective cohort study, a higher eCRF was associated with a lower MI risk in both females and males and among those with other CVD risk factors, but associations were more pronounced among females than that in males. These findings suggest eCRF is a vital estimate to implement in routine medical care to identify individuals at high risk of future MI, especially for females.

## Potential Competing Interests

M.L.L. has received lecture fees from 10.13039/100004326Bayer, 10.13039/100004339Sanofi, and 10.13039/100002491BMS/10.13039/100004319Pfizer not related to this study. The remaining authors declare no conflict of interest.
