# Supplementary material for: Females Display Lower Risk of Myocardial Infarction From Higher Estimated Cardiorespiratory Fitness Than Males: The Tromsø Study 1994-2014
Source: Mayo Clin Proc Innov Qual Outcomes. 2024 Jan 6;8(1):62–73. doi: 10.1016/j.mayocpiqo.2023.12.007 (PMC10806283; doi:10.1016/j.mayocpiqo.2023.12.007)
Supplement: Supplementary materials [file mmc1.docx]

**Supplementary materials**

**Contents**

[**Supplementary File S1.** The estimation of cardiorespiratory fitness. 3](#_Toc150725010)

[**Supplementary Table S1.** The Cohort of Norway Physical Activity questionnaire*. 3](#_Toc150725011)

[**Supplementary Table S2.** The physical activity index derived from the Physical Activity Frequency, Intensity, and Duration questionnaire*. 4](#_Toc150725012)

[**Supplementary File S2.** Harmonization of education 4](#_Toc150725013)

[**Supplementary File S3.** Description of alcohol intake. 5](#_Toc150725014)

[**Supplementary Table S3.** Questions and answering alternatives for alcohol intake in Tromsø4 (1994-95). 5](#_Toc150725015)

[**Supplementary Table S4.** Questions and answering alternatives for alcohol intake in Tromsø5 (2001). 5](#_Toc150725016)

[**Supplementary Table S5.** Questions and answering alternatives for alcohol intake in Tromsø6 (2007-08) and in Tromsø7 (2015-16). 6](#_Toc150725017)

[Supplementary File S4. Harmonization of diet quality 6](#_Toc150725018)

[**Supplementary Table S6.** Questions, answer alternatives and calculation of diet quality in Tromsø4 (1994-95). 7](#_Toc150725019)

[**Supplementary Table S7.** Questions, answer alternatives and calculation of diet quality in Tromsø5 (2001). 9](#_Toc150725020)

[**Supplementary Table S8.** Questions, answer alternatives and calculation of diet quality in Tromsø6 (2007-08). 10](#_Toc150725021)

[**Supplementary File S5.** Harmonization of traditional risk factors for cardiovascular disease 11](#_Toc150725022)

[**Supplementary Table S7.** Calculation of metabolic equivalent of tasks in the Cohort of Norway physical activity questionnaire. 12](#_Toc150725023)

[**Supplementary Table S8**. Calculation of metabolic equivalent of tasks in the physical activity frequency, intensity, and duration questionnaire. 12](#_Toc150725024)

[**Supplementary Table S9.** Survey-specific descriptive characteristics of the participants. The Tromsø Study 1994-2014. 13](#_Toc150725025)

[**Supplementary Table S10.** The association between estimated cardiorespiratory fitness and myocardial infarction among participants under and over 60 years. The Tromsø Study 1994-2014. 14](#_Toc150725026)

[**Supplementary Table S11.** The association between estimated cardiorespiratory fitness and myocardial infarction in age-standardized quintiles among females. The Tromsø Study 1994-2014. 15](#_Toc150725027)

[**Supplementary Table S12.** The association between estimated cardiorespiratory fitness and myocardial infarction in age-standardized quintiles among males. The Tromsø Study 1994-2014. 15](#_Toc150725028)

[**Supplementary Table S13. The association between estimated cardiorespiratory fitness and myocardial infarction in those with ≥5 years follow-up time. The Tromsø Study 1994-2014.** 16](#_Toc150725029)

[**Supplementary Figure S1. Directed acyclic graph for the hypothesized association.** 17](#_Toc150725030)

[**Supplementary Figure S2.** Log-log survival plot of subdistributed hazards of smoking in females. 18](#_Toc150725031)

[**Supplementary Figure S3.** Log-log survival plot of subdistributed hazards of smoking in males. 19](#_Toc150725032)

[**Supplementary Figure S4.** Flow chart of included participants 20](#_Toc150725033)

[**References for the supplementary materials** 20](#_Toc150725034)

# **Supplementary File S1.** The estimation of cardiorespiratory fitness.

In both formulas, specific index scores for physical activity are used for eCRF (1, 2). The CONOR questionnaire asks how many weekly hours one spends doing physical activity in light (not out of breath) and hard (out of breath) intensity, with answer alternatives: 1) none; 2) under one hour; 3) 1-2 hours; 4) ≥3 hours (3) (Supplementary Table S1). For the formula by Nauman *et al.* (1), physical activity was given value 1 if participants reported being active for more than three weekly hours; *i.e.*, rank 4 in either light or hard or the summed product exceeded 3 hours, slightly over the current lower-limit physical activity guidelines of 150 minutes per week of moderate and vigorous physical activity (4), otherwise given the value 0 (Supplementary Table S1). The PAFID asks about frequency, intensity, and duration of physical activity, where the index was given as the product of frequency, intensity, and duration according to Nes *et al.* (2) (Supplementary Table S2).

# **Supplementary Table S1.** The Cohort of Norway Physical Activity questionnaire*.

| **Question** | **Answer alternatives** |
| --- | --- |
| *How has your physical activity in leisure time been during this last year? Think of your weekly average for the year (hours per week)* |  |
| *Light activity (not sweating or out of breath)* | 1: None (0)  2: Less than 1 hour (0.5)  3: 1-2 hours (1.5)  4: 3 or more hours (3) |
| *Hard physical activity (sweating/out of breath)* | 1: None (0)  2: Less than 1 hour (0.5)  3: 1-2 hours (1.5)  4: 3 or more hours (3) |

*Numbers in parentheses indicate hours for calculation of hours of physical activity.

# **Supplementary Table S2.** The physical activity index derived from the Physical Activity Frequency, Intensity, and Duration questionnaire*.

| **Frequency (days)** | **Intensity (METs)** | **Duration (minutes)** |
| --- | --- | --- |
| *How frequently do you exercise? With exercise, we mean walking, cross-country skiing, swimming or other exercise/sports*. | *On average, how hard is the exercise?* | *On average, how long do you exercise?* |
| Never (0) | I take it easy without breaking into a sweat or loosing my breath (0) | <15 minutes (1) |
| Less than once a week (0) | I push myself so hard that I break into a sweat and loose my breath (5) | 15-29 minutes (1) |
| Once a week (1) | I push myself to near-exhaustion (10) | 30-60 minutes (1.5) |
| 2-3 times per week (2) | N/A | >60 minutes (1.5) |
| Almost every day (3) | N/A | N/A |

*Numbers in parentheses was multiplied to display the physical activity index used by Nes *et al.* (26).

# **Supplementary File S2.** Harmonization of education

Information on education was retrieved from questionnaires. In Tromsø6 (2007-08) and Tromsø7 (2015-16), participants were asked for education in four groups 1) Primary school, 2) High School, 3) University <4 years and 4) University ≥4 years. In Tromsø3 (1986-87) and Tromsø5 (2001), participants were asked about years of education, which we categorized as the four abovementioned groups according to the Norwegian educational system, as: 1) Primary School, <10 years education; 2) High School, 10-12 years; 3) University <4 years, 13-15 years; 4) University ≥4 years, ≥16 years. In Tromsø4 (1994-95), participants answered education as five groups, the four abovementioned groups and a fifth group, Technical/vocational school, which we categorized as 2) High School.

# **Supplementary File S3.** Description of alcohol intake.

Alcohol intake was retrieved from questionnaires. Questions on alcohol have been changing across surveys. In Tromsø4 (1994-95), participants answered questions on frequency of beer, wine and spirits intake, a question on frequency of binge drinking, and if being a teetotaler. Questions in Tromsø 5-6 also included questions on volume of alcohol intake. To harmonize alcohol intake across surveys, we considered the frequency in Tromsø4 to include one units of alcohol, equivalent to Norwegian standards of alcohol units: 1 bottle of 0.33 L beer, 1.5 dl glass of wine and 4 cl of spirits. Answers on all questions were summed as units per week. The calculation of all questions in each survey to represents units per week are described in Supplementary Table S1-3.

## **Supplementary Table S3.** Questions and answering alternatives for alcohol intake in Tromsø4 (1994-95).

| ***Question*** | **Answers*** |
| --- | --- |
| *Are you a teetotaller?* | 1: Yes (0)  2: No (N/A) |
| *How many glasses of beer do you normally drink in a fortnight. Do not*  *count low-alcohol beer. Put 0 if less than once a month.* | Continuous scale |
| *How many glasses of wine do you normally drink in a fortnight. Put 0 if less than once a month.* | Continuous scale |
| *How many glasses of spirits do you normally drink in a fortnight. Put 0 if less than once a month.* | Continuous scale |

*Frequency was regarded as one unit, which was summed to total units per week.

## **Supplementary Table S4.** Questions and answering alternatives for alcohol intake in Tromsø5 (2001).

| ***Question*** | **Answers*** |
| --- | --- |
| *Are you a teetotaller?* | 1: Yes (0)  2: No (N/A) |
| *Approximately, how often have you during the last year consumed alcohol (do not count low-alcohol beer)?* | 1: Never consumed alcohol (0)  2: Not during the last year (0)  3: A few times (0.125)  4: 1 time per month (0.25)  5: 2-3 times per month (0.65)  6: 1 time per week (1)  7: 2-3 times per week (2.5)  8: 4-7 times per week (5.5) |
| *When you drink alcohol, how many glasses or drinks do you normally drink?* | Continuous scale multiplied by number in parentheses of question on frequency. |
| *Approximately how many times during the last year have you consumed alcohol equivalent to 5 glasses or drinks within 24 hours.* | Continuous scale divided by 52 weeks |

*Numbers in parentheses indicate times per week, which was multiplied with numbers of drink when they usually drank (continuous scale). This was summed with the question of binge drinking.

## **Supplementary Table S5.** Questions and answering alternatives for alcohol intake in Tromsø6 (2007-08) and in Tromsø7 (2015-16).

| ***Question*** | **Answers*** |
| --- | --- |
| *Are you a teetotaller?* | 1: Yes (0)  2: No (N/A) |
| *How often do you usually drink alcohol?* | 1: Never (0)  2: Monthly or less frequently (0.25)  3: 2-4 times a month (1)  4: 2-3 times a week (2.5)  4: 4 or more times a week (5.5) |
| *How many units of alcohol (a beer, a glass of wine or a drink) do you*  *usually drink when you drink alcohol?* | 1: 1-2 (1.5)  2: 3-4 (3.5)  3: 5-6 (5.5)  4: 7-9 (8)  5: 10 or more (10) |
| *How often do you drink 6 units alcohol or more in one occasion?* | 1: Never (0)  2: Less frequently than monthly (1.5)  3: Monthly (1.5)  4: Weekly (6)  5: Daily or almost daily (30) |

*Numbers in parentheses indicate times per week for the frequency question and units for the volume question, which was multiplied to units per week. Number in parentheses for the binge drinking question indicate units per week, which was summed with units per week from combined frequency and volume question.

## Supplementary File S4. Harmonization of diet quality

Diet quality was retrieved from questionnaires, where questions were harmonized to display number of national nutrition guidelines for fruit/vegetables/berries, fish, processed meat and saturated fat intake (5) that participants met. The number of questions and possible nutrition guidelines to meet differed across surveys due to number of included questions. The harmonization is shown in Supplementary Tables S4-6.

## **Supplementary Table S6.** Questions, answer alternatives and calculation of diet quality in Tromsø4 (1994-95).

| **Nutritional guideline** | **Question** | Answer alternatives* |
| --- | --- | --- |
| *Fish intake* |  |  |
| *“Eat fish 2-3 times per week”* | *How many times per week do you normally eat fat fish (e.g. salmon/redfish) for dinner?* | 1: Never (0)  2: <1 (0.2)  3: 1 (0.3)  4: 2-3 (1)  5: 4-5 (1)  6: Approximately every day (1) |
| *“Eat fish 2-3 times per week”* | *How many times per week do you normally eat lean fish (e.g. cod) for dinner?* | 1: Never (0)  2: <1 (0.2)  3: 1 (0.3)  4: 2-3 (1)  5: 4-5 (1)  6: Approximately every day (1) |
| *“Eat fish 2-3 times per week”* | *How many slices of bread with fish (e.g. mackerel in tomato sauce) do you usually eat daily (number)?* | 1: 0 (0)  2: <1 (0.05)  3: 1-2 (0.1)  4: 3-4 (0.15)  5: 5-6 (0.2)  6: >6 (0.3) |
| *Saturated fat intake* |  |  |
| *“Choose oils, liquid or soft margarine.”* | *What type of margarine or butter do you usually use on your bread?* | 1: Do not use margarine or butter on bread (1)  2: Butter (0)  3: Hard margarine (0)  4: Soft margarine (1)  5: Butter/margarine mixtures (0.5)  6: Light margarine (1) |
| *“Choose oils, liquid or soft margarine.”* | *Do you normally use butter in cooking (not on the bread) in your*  *home?* | 1: Yes (0) |
| *“Choose oils, liquid or soft margarine.”* | *Do you normally use hard margarine in cooking (not on the bread) in your home?* | 1: Yes (0) |
| *“Choose oils, liquid or soft margarine.”* | *Do you normally use hard margarine in cooking (not on the bread) in your home?* | 1: Yes (0) |
| *“Choose oils, liquid or soft margarine.”* | *Do you normally use soft margarine in cooking (not on the bread) in your home?* | 1: Yes (1) |
| *“Choose oils, liquid or soft margarine.”* | *Do you normally use butter/margarine blend in cooking (not on the bread) in your home?* | 1: Yes (0) |
| *“Choose oils, liquid or soft margarine.”* | *Do you normally use oils in cooking in your home?* | 1: Yes (1) |
|  |  | If answering yes on more than one of use of butter/margarine/oil in cooking, the value 0.5 is given (*e.g.*, if using both oils (1) and hard margarine (0) = 0.5) |
| *Fruit/vegetables intake* |  |  |
| *“Eat five portions of fruit, vegetables and/or berries each day.”* | *How many times per week do you normally eat vegetables for dinner?* | 1: Never (0)  2: <1 (0.03)  3: 1 (0.1)  4: 2-3 (0.1)  5: 4-5 (0.15)  6: Approximately every day (0.2) |
| *“Eat five portions of fruit, vegetables and/or berries each day.”* | *How many times per week do you normally eat apples/pears?* | 1: Never (0)  2: <1 (0.03)  3: 1 (0.1)  4: 2-3 (0.1)  5: 4-5 (0.15)  6: Approximately every day (0.2) |
| *“Eat five portions of fruit, vegetables and/or berries each day.”* | *How many times per week do you normally eat oranges, mandarines?* | 1: Never (0)  2: <1 (0.03)  3: 1 (0.1)  4: 2-3 (0.1)  5: 4-5 (0.15)  6: Approximately every day (0.2) |
| *“Eat five portions of fruit, vegetables and/or berries each day.”* | *How much orange juice do you usually drink daily (glasses) ?* | 1: 0 (0)  2: <1 (0)  3: 1-2 (0.25)  4: 3-4 (0.6)  5: 5-6 (1)  6: >6 (1) |
| Processed meat intake |  |  |
| *“Choose non-processed meat and limit intake of red meat.”* | *How many slices of bread with fat meat (e.g. salami) do you usually eat daily (number)?* | 1: 0 (1)  2: <1 (1)  3: 1-2 (0.5)  4: 3-4 (0.25)  5: 5-6 (0)  6: >6 (0) |
| *“Choose non-processed meat and limit intake of red meat.”* | *How many times per week do you normally eat unprocessed meat for dinner?* | 1: Never (1)  2: <1 (1)  3: 1 (0.25)  4: 2-3 (0)  5: 4-5 (0)  6: Approximately every day (0) |
| *“Choose non-processed meat and limit intake of red meat.”* | *How many times per week do you normally eat sausage/meatloaf/meatballs for dinner?* | 1: Never (1)  2: <1 (1)  3: 1 (0.25)  4: 2-3 (0)  5: 4-5 (0)  6: Approximately every day (0) |

*Each nutritional guideline was summed to represent whether it was met. If obtaining higher than 1 for one guideline, the number was replaced with 1 (e.g., if obtaining 2 from the fish intake questions, it was replaced with 1 as one cannot met a guideline twice per week). Each guideline was thereafter summed. In Tromsø4 (1994-95), number of guidelines met ranged from 0.0-4.0.

## **Supplementary Table S7.** Questions, answer alternatives and calculation of diet quality in Tromsø5 (2001).

| **Nutritional guideline** | **Question** | Answer alternatives* |
| --- | --- | --- |
| *Fish intake* |  |  |
| *“Eat fish 2-3 times per week”* | *How often do you usually eat fat fish (e.g. salmon, trout, mackerel, herring)?* | 1: Rarely/never (0)  2: 1-3 times per month (0.15)  3: 1-3 times per week (1)  4: 4-6 times per week (1)  5: 1-2 times per day (1)  6: 3 or more times per day (1) |
| *“Eat fish 2-3 times per week”* | *Do you use cod liver oil or fish oil capsules?* | 1: Yes, daily (1)  2: Sometimes (0.5)  3: No (0) |
| *Saturated fat intake* |  |  |
| *“Choose oils, liquid or soft margarine.”* | *What type of fat do you usually use for cooking?* | 1: Do not use margarine or butter (1)  2: Butter (0)  3: Hard margarine (0)  4: Soft/light margarine (1)  5: Oils (1)  6: Other (0) |
| *“Choose oils, liquid or soft margarine.”* | *What type of fat do you usually use on your bread?* | 1: Do not use margarine or butter (1)  2: Butter (0)  3: Hard margarine (0)  4: Soft/light margarine (1)  5: Oils (1)  6: Other (0) |
|  |  | If answering an alternative with value 1 for cooking and 0 for bread, the value 0.5 is given (*e.g.*, if using both oils (1) for cooking but hard margarine (0) margarine for bread = 0.5) |
| *Fruit/vegetables intake* |  |  |
| *“Eat five portions of fruit, vegetables and/or berries each day.”* | *How often do you usually eat fruit and berries?* | 1: Rarely/never (0)  2: 1-3 times per month (0)  3: 1-3 times per week (0.075)  4: 4-6 times per week (0.15)  5: 1-2 times per day (0.3)  6: 3 or more times per day (0.6) |
| *“Eat five portions of fruit, vegetables and/or berries each day.”* | *How often do you usually eat fresh vegetables/salad?* | 1: Rarely/never (0)  2: 1-3 times per month (0)  3: 1-3 times per week (0.075)  4: 4-6 times per week (0.15)  5: 1-2 times per day (0.3)  6: 3 or more times per day (0.6) |
| *“Eat five portions of fruit, vegetables and/or berries each day.”* | *Do you use vitamins and/or mineral supplement?* | 1: Yes, daily (1)  2: Sometimes (0.5)  3: No (0) |
| *“Eat five portions of fruit, vegetables and/or berries each day.”* | *How much juice do you normally drink?* | 1: Rarely/never (0)  2: 1-6 glasses per week (0.15)  3: 1 glass per day (0.2)  4: 2-3 glasses per day (0.5)  5: 4 or more glasses per day (0.8) |

*Each nutritional guideline was summed to represent whether it was met. If obtaining higher than 1 for one guideline, the number was replaced with 1 (e.g., if obtaining 2 from the fish intake questions, it was replaced with 1 as one cannot met a guideline twice per week). Each guideline was thereafter summed. In Tromsø5 (2001), number of guidelines met ranged from 0.0-3.0.

## **Supplementary Table S8.** Questions, answer alternatives and calculation of diet quality in Tromsø6 (2007-08).

| **Nutritional guideline** | **Question** | Answer alternatives* |
| --- | --- | --- |
| *Fish intake* |  |  |
| *“Eat fish 2-3 times per week”* | *How often do you usually eat fat fish (e.g. salmon, trout, mackerel, herring, halibut, redfish) for dinner?* | 1: 0-1 times per month (0)  2: 2-3 times per month (0.2)  3: 1-3 times per week (1)  4: 4-6 times per week (1)  5: 1-2 times per day (1) |
| *“Eat fish 2-3 times per week”* | *Do you use cod liver oil or fish oil capsules?* | 1: Yes, daily (1)  2: Sometimes (0.5)  3: No (0) |
| *“Eat fish 2-3 times per week”* | *Do you use Omega 3 capsules (fish oil, seal oil)?* | 1: Yes, daily (1)  2: Sometimes (0.5)  3: No (0) |
| *Fruit/vegetables intake* |  |  |
| *“Eat five portions of fruit, vegetables and/or berries each day.”* | *How often do you usually eat fruits, vegetables and berries?* | Continuous scale,  0 (0), 1 (0.2), 2 (0.4), 3 (0.6), 4 (0.8), ≥5 (1). |
| *“Eat five portions of fruit, vegetables and/or berries each day.”* | *How many units of fruit or vegetables do you eat per day (average).*  *(E.g. a fruit, a cup of juice, potatoes, vegetables)* | 1: 0-1 times per month (0)  2: 2-3 times per month (0)  3: 1-3 times per week (0.075)  4: 4-6 times per week (0.15)  5: 1-2 times per day (0.3) |
| *“Eat five portions of fruit, vegetables and/or berries each day.”* | *How much juice do you normally drink?* | 1: Rarely/never (0)  2: 1-6 glasses per week (0.15)  3: 1 glass per day (0.2)  4: 2-3 glasses per day (0.5)  5: 4 or more glasses per day (0.8) |
| *“Eat five portions of fruit, vegetables and/or berries each day.”* | *Do you use vitamins and/or mineral supplement?* | 1: Yes, daily (1)  2: Sometimes (0.5)  3: No (0) |

*Each nutritional guideline was summed to represent whether it was met. If obtaining higher than 1 for one guideline, the number was replaced with 1 (*e.g.*, if obtaining 2 from the fish intake questions, it was replaced with 1 as one cannot met a guideline twice per week). Each guideline was thereafter summed. In Tromsø6 (2007-08), number of guidelines met ranged from 0.0-2.0.

# **Supplementary File S5.** Harmonization of traditional risk factors for cardiovascular disease

Hypertension was dichotomized as yes/no and was retrieved from questionnaires: Tromsø4-5 (1994-95, 2001): *“Do you use blood pressure lowering drugs?”*; Tromsø6 (2008-07: *“Have you ever had, or do you have high blood pressure?”*. To avoid misclassification of hypertension, we also used ATC-codes (C02,03, C07, C08, C09, link: <https://www.whocc.no/>) from reported preparation names of medication used by the participants the last four weeks and used measured systolic (>130 mmHg) and diastolic (>85 mmHg) blood pressure at study attendance, which was measured three times in a seated position, where we used the mean of the last two recordings.

Hyperlipidaemia was dichotomized as yes/no, which we retrieved from blood samples of serum total cholesterol (≥5.17 mmol/L), and if using lipid lowering drugs (from question *“Do you use cholesterol lowering drugs?”*) and reported use last four weeks medication with ATC-code C10.

Central obesity was defined as waist circumference-thresholds (cm) at specific BMI-thresholds (kg/m^2^) according to Ross *et al* (6); females: <25 kg/m^2^+≥80 cm, 25-29 kg/m^2^+≥90 cm, 30-34 kg/m^2^+≥105 cm, BMI=≥35 kg/m^2^+≥115 cm; males: <25 kg/m^2^+≥90 cm, 25-29 kg/m^2^+≥100 cm, 30-34 kg/m^2^+≥ 110 cm, ≥35 kg/m^2^+≥125 cm. Measurements of waist circumference is described above (Supplementary File S1), and BMI was calculated from measured weight and height in light clothing, given as kg/m^2^. Metabolic syndrome was defined according to the International Federation of Diabetes (7); waist circumference: females: ≥88 cm; males: ≥94 cm, and/or BMI≥30 kg/m^2^, in addition to two of the following: 1) triglyceride levels: ≥1.7 mmol/L, or if using lipid lowering drugs; 2) high-density lipoprotein: <1.03 mmol/L for males, 50 mmol/L for females; 3) hypertension (described above), or 4) diagnosed with diabetes (Tromsø4-6 did not include fasting glucose measurements). Diabetes information was retrieved from 1) questionnaire (*“Do you have, or have you had diabetes?”*); and/or 2) using blood sugar lowering drugs and/or insulin now; and/or 3) reporting use of preparations containing insulin and /or blood sugar lowering medication (ATC=A10A and/or A10B).

# **Supplementary Table S7.** Calculation of metabolic equivalent of tasks in the Cohort of Norway physical activity questionnaire.

| **Question** | **Answer alternatives** |
| --- | --- |
| *How has your physical activity in leisure time been during this last year? Think of your weekly average for the year (hours per week)* | Parentheses illustrate hours for calculation |
| *Light activity (not sweating or out of breath)* | 1: None (0 x 3 METs)  2: Less than 1 hour (0.5 x 3 METs)  3: 1-2 hours (1.5 x 3 METs)  4: 3 or more hours (3 x 3 METs) |
| *Hard physical activity (sweating/out of breath)* | 1: None (0 x 6 METs)  2: Less than 1 hour (0.5 x 6 METs)  3: 1-2 hours (1.5 x 6 METs)  4: 3 or more hours (3 x 6 METs) |

*MET-hours per week is given by addition of METs in light and hard activity. MET=metabolic equivalent of task.

# **Supplementary Table S8**. Calculation of metabolic equivalent of tasks in the physical activity frequency, intensity, and duration questionnaire.

| **Frequency (days)** | **Intensity (METs)** | **Duration (minutes)** |
| --- | --- | --- |
| *How frequently do you exercise? With exercise, we mean walking, cross-country skiing, swimming or other exercise/sports*. | *On average, how hard is the exercise?* | *On average, how long do you exercise?* |
| Never (0) | I take it easy without breaking into a sweat or loosing my breath (3) | <15 minutes (10) |
| Less than once a week (0.5) | I push myself so hard that I break into a sweat and loose my breath (6) | 15-29 minutes (22.5) |
| Once a week (1) | I push myself to near-exhaustion (9) | 30-60 minutes (45) |
| 2-3 times per week (2.5) | N/A | >60 minutes (60) |
| Almost every day (5) | N/A | N/A |

*Numbers in parentheses were multiplied to obtain METs as *“(duration x intensity) x frequency”* according to Sagelv *et al.* (8).

# **Supplementary Table S9.** Survey-specific descriptive characteristics of the participants. The Tromsø Study 1994-2014.

|  | **Total cohort** | **Tromsø4 1994-95** | **Tromsø5 2001** | **Tromsø6 2007-08** |
| --- | --- | --- | --- | --- |
| **n** | 14285 | 5282 | 2692 | 6311 |
| Myocardial infarction, n (%) | 979 (6.9) | 811 (15.4) | 97 (3.9) | 71 (1.1) |
| Incidence per 1000, rate (95%CI) | 7.6 (7.2-9.1) | 11.1 (10.4-11.9) | 3.5 (2.9-4.3) | 2.6 (2.0-3.2) |
| **Follow-up time (years)** |  |  |  |  |
| Median (25^th^-75^th^ percentile) | 7.2 (6.3-14.6) | 19.6 (11.9-19.9) | 13.2 (13.1-13.5) | 6.3 (6.2-6.8) |
| Min-max | 2.0-20.3 | 2.0-20.3 | 2.0-13.8 | 2.0-7.2 |
| **Sex** |  |  |  |  |
| Females, n (%) | 7873 (55.1) | 2819 (53.4) | 1753 (65.1) | 3301 (52.3) |
| Males, n (%) | 6412 (44.9) | 2463 (46.6) | 939 (34.9) | 3010 (47.7) |
| **Age (years), mean ± SD** | 53.7 ± 11.4 | 59.2 ± 10.4 | 48.4 ± 12.5 | 51.3 ± 9.8 |
| <30 years, n (%) | 589 (4.1) | 123 (2.3) | 437 (16.2) | 29 (0.5) |
| 30-39 years, n (%) | 1632 (11.4) | 265 (5.0) | 579 (21.5) | 788 (12.5) |
| 40-49 years, n (%) | 3277 (22.9) | 459 (8.7) | 518 (19.2) | 2300 (36.4) |
| 50-59 years, n (%) | 4445 (31.1) | 1856 (35.1) | 808 (30.0) | 1781 (28.2) |
| 60-69 years, n (%) | 3550 (24.9) | 1992 (37.7) | 245 (9.1) | 1313 (20.8) |
| ≥70 years, n (%) | 792 (5.5) | 587 (11.1) | 105 (3.9) | 100 (1.6) |
| **Education** |  |  |  |  |
| Primary school, n (%) | 4608 (32.3) | 2697 (51.1) | 774 (28.8) | 1137 (18.0) |
| High School, n (%) | 4504 (31.5) | 1604 (30.4) | 744 (27.6) | 2156 (34.2) |
| University <4 years, n (%) | 2372 (16.6) | 568 (10.8) | 501 (18.6) | 1303 (20.7) |
| University ≥4 years, n (%) | 2801 (19.6) | 413 (7.8) | 673 (25.0) | 1715 (27.2) |
| **Adiposity markers** |  |  |  |  |
| Body mass index (kg/m^2^), mean ± SD | 26.3 ± 4.2 | 25.7 ± 3.9 | 26.2 ± 4.3 | 26.7 ± 4.2 |
| <25 kg/m^2^, n (%) | 5953 (41.7) | 2376 (45.0) | 1195 (44.4) | 2382 (37.7) |
| 25-29 kg/m^2^, n (%) | 6044 (42.3) | 2218 (42.0) | 1052 (39.1) | 2774 (44.0) |
| ≥30 kg/m^2^, n (%) | 2281 (16.0) | 683 (12.9) | 443 (16.5) | 1155 (18.3) |
| Central obesity, n (%)# | 4647 (32.5) | 1374 (26.0) | 608 (22.6) | 2665 (42.2) |
| **Diet quality (nutritional guideline), mean ± SD** | 1.7 ± 0.7 | 2.0 ± 0.9 | 1.5 ± 0.8 | 1.6 ± 0.5 |
| <1 nutritional guideline, n (%) | 3463 (24.3) | 1581 (29.9) | 810 (30.1) | 1072 (17.0) |
| 1-2 nutritional guideline, n (%) | 7347 (51.4) | 921 (17.4) | 1187 (44.1) | 5239 (83.0) |
| ≥2 nutritional guideline, n (%) | 3475 (24.3) | 2780 (52.6) | 695 (25.8) | N/A |
| **Alcohol intake (mean ± SD)** | 2.5 ± 3.2 | 1.7 ± 2.9 | 2.3 ± 3.1 | 3.3 ± 3.3 |
| Teetotaller, n (%) | 2974 (20.8) | 2361 (44.7) | 330 (12.3) | 283 (4.5) |
| 0.1-1.9 units∙week^-1^, n (%) | 6139 (43.0) | 1589 (30.1) | 1518 (56.4) | 3032 (48.0) |
| 2.0-3.9 units∙week^-1^, n (%) | 2880 (20.2) | 716 (13.6) | 367 (13.6) | 1797 (28.5) |
| ≥ 4.0 units∙week^-1^, n (%) | 2292 (16.0) | 616 (11.7) | 477 (17.7) | 1199 (19.0) |
| **Smoking** |  |  |  |  |
| Current smoker, n (%) | 3823 (26.8) | 1701 (32.2) | 874 (32.5) | 1248 (19.8) |
| Previous smoker, n (%) | 5219 (36.5) | 1843 (34.9) | 830 (30.8) | 2546 (40.3) |
| Never smoker, n (%) | 5243 (36.7) | 1738 (32.9) | 988 (36.7) | 2517 (39.9) |
| **Physical activity (MET-hours∙week^-1^), mean ± SD** | 9.4 ± 7.5 | 8.7 ± 6.9 | 11.0 ± 7.3 | 9.2 ± 7.9 |
| <7.5 MET-hours∙week^-1^, n (%) | 6255 (43.8) | 2213 (41.9) | 775 (28.8) | 3267 (51.8) |
| <7.5-15 MET-hours∙week^-1^, n (%) | 5603 (39.2) | 2191 (41.5) | 1215 (45.1) | 2197 (34.8) |
| ≥15 MET-hours∙week^-1^, n (%) | 2427 (17.0) | 878 (16.6) | 702 (26.1) | 847 (13.4) |
| **eCRF (mL∙kg^-1^∙min^-1^), mean ± SD** | 36.3 ± 8.0 | 33.9 ± 7.4 | 37.5 ± 8.2 | 37.7 ± 8.1 |
| **Disease, n** (%) | 632 (4.4) | 366 (6.9) | 68 (2.5) | 198 (3.1) |
| Cancer, n (%) | 365 (2.6) | 243 (2.3) | 63 (2.3) | 59 (0.9) |
| Diabetes, n (%) | 397 (2.8) | 129 (2.4) | 69 (2.6) | 199 (3.2) |
| **Ill condition** |  |  |  |  |
| Hypertension, n (%) | 8290 (58.0) | 3803 (72.0) | 1214 (45.1) | 3273 (51.9) |
| Hyperlipidaemia, n (%) | 2956 (20.7) | 2052 (38.9) | 568 (21.2) | 336 (5.3) |

Data are shown as frequency (%) or as mean ± SD. *quartiles of eCRF of the total cohort. #central obesity includes those with ≥30 kg/m^2^ and/or those ≥102 and ≥88 cm in waist circumference for males and females, respectively. eCRF=estimates cardiorespiratory fitness, MET=metabolic equivalent of task, SD=standard deviation.

# **Supplementary Table S10.** The association between estimated cardiorespiratory fitness and myocardial infarction among participants under and over 60 years. The Tromsø Study 1994-2014.

| **<60 years** | **Females** | **Males** |
| --- | --- | --- |
|  | *N (MI)* | *N (MI)* |
|  | *5529 (141)* | *4414 (258)* |
| Arbitrary eCRF values | SHR (95%CI) | SHR (95%CI) |
| 21 mL∙kg^-1^∙min^-1^ | 1.13 (0.89-1.44) | N/A |
| 25 mL∙kg^-1^∙min^-1^ | *Ref.* | N/A |
| 30 mL∙kg^-1^∙min^-1^ | 0.84 (0.63-1.12) | 1.05 (0.98-1.13) |
| 32 mL∙kg^-1^∙min^-1^ | 0.72 (0.50-1.04) | *Ref.* |
| 35 mL∙kg^-1^∙min^-1^ | **0.45 (0.29-0.71)** | 0.93 (0.83-1.04) |
| 40 mL∙kg^-1^∙min^-1^ | **0.10 (0.03-0.34)** | 0.81 (0.61-1.06) |
| 45 mL∙kg^-1^∙min^-1^ | **0.02 (0.00-0.19)** | **0.66 (0.47-0.94)** |
| 50 mL∙kg^-1^∙min^-1^ | **0.001 (0.001-0.12)** | **0.51 (0.32-0.81)** |
| 55 mL∙kg^-1^∙min^-1^ | N/A | **0.39 (0.19-0.80)** |
| 60 mL∙kg^-1^∙min^-1^ | N/A | **0.29 (0.10-0.84)** |
| **≥60 years** | **Females** | **Males** |
|  | *N (MI)* | *N (MI)* |
|  | *2842 (295)* | *2423 (338)* |
| Arbitrary eCRF values | SHR (95%CI) | SHR (95%CI) |
| 20 mL∙kg^-1^∙min^-1^ | 1.18 (0.91-1.54) | N/A |
| 25 mL∙kg^-1^∙min^-1^ | *Ref.* | 1.26 (0.96-1.66) |
| 30 mL∙kg^-1^∙min^-1^ | **0.64 (0.53-0.78)** | 1.07 (0.99-1.15) |
| 32 mL∙kg^-1^∙min^-1^ | **0.48 (0.34-0.68)** | *Ref.* |
| 35 mL∙kg^-1^∙min^-1^ | **0.30 (0.16-0.57)** | 0.91 (0.83-0.99) |
| 40 mL∙kg^-1^∙min^-1^ | **0.19 (0.07-0.49)** | **0.78 (0.65-0.93)** |
| 45 mL∙kg^-1^∙min^-1^ | N/A | **0.67 (0.46-0.99)** |
| 50 mL∙kg^-1^∙min^-1^ | N/A | 0.58 (0.31-1.10) |

Data are adjusted for education, diet, smoking, alcohol intake, and study survey. Values are arbitrary values of the restricted cubic spline models. eCRF=estimated cardiorespiratory fitness. SHR=subdistributed hazard ratio, CI=confidence interval, Mi=myocardial infarction. Bold number indicate significant difference from reference category.

# **Supplementary Table S11.** The association between estimated cardiorespiratory fitness and myocardial infarction in age-standardized quintiles among females. The Tromsø Study 1994-2014.

|  | **Quintile 1** | **Quintile 2** | **Quintile 3** | **Quintile 4** | **Quintile 5** |
| --- | --- | --- | --- | --- | --- |
| *<40 years* | *252 (3)* | *252 (0)* | *254 (0)* | *240 (1)* | *247 (0)* |
| eCRF (mL∙kg^-1^∙min^-1^) | 32.5 ± 2.6 | 36.8 ± 0.7 | 39.0 ± 0.7 | 41.3 ± 0.7 | 44.9 ± 2.4 |
| *40-49 years* | *349 (2)* | *354 (2)* | *339 (0)* | *327 (1)* | *341 (1)* |
| eCRF (mL∙kg^-1^∙min^-1^) | 29.5 ± 2.5 | 33.7 ± 0.7 | 35.9 ± 0.7 | 38.1 ± 0.7 | 43.3 ± 4.0 |
| *50-59 years* | *521 (40)* | *521 (25)* | *507 (33)* | *513 (25)* | *511 (8)* |
| eCRF (mL∙kg^-1^∙min^-1^) | 25.2 ± 2.3 | 29.2 ± 0.7 | 31.3 ± 0.6 | 33.2 ± 0.6 | 37.3 ± 3.4 |
| *60-69 years* | *381 (56)* | *374 (53)* | *381 (48)* | *367 (25)* | *366 (12)* |
| eCRF (mL∙kg^-1^∙min^-1^) | 22.4 ± 2.2 | 26.2 ± 0.7 | 28.2 ± 0.6 | 30.1 ± 0.6 | 34.0 ± 2.8 |
| *≥70 years* | *98 (21)* | *92 (12)* | *96 (15)* | *96 (16)* | *93 (21)* |
| eCRF (mL∙kg^-1^∙min^-1^) | 19.4 ± 2.1 | 22.7 ± 0.7 | 24.7 ± 0.6 | 26.5 ±0.6 | 29.4 ± 1.9 |
| *Total, n (mi)* | *1601 (122)* | *1593 (92)* | *1577 (96)* | *1543 (68)* | *1559 (42)* |
| SHR (95%CI) | Ref. | **0.72 (0.55-94)** | 0.80 (0.61-1.04) | **0.59 (0.44-0.80)** | **0.58 (0.41-0.83)** |

Data are adjusted for education, diet quality, smoking, alcohol intake, study survey, and age as timescale. Number of participants (MI) and their mean ± SD of eCRF are shown per 10-year age group, and as merged N (mi). eCRF=estimated cardiorespiratory fitness. SHR=subdistributed hazard ratio, CI=confidence interval, MI?...... SD=standard deviation. Bold number indicate significant association.

# **Supplementary Table S12.** The association between estimated cardiorespiratory fitness and myocardial infarction in age-standardized quintiles among males. The Tromsø Study 1994-2014.

|  | **Quintile 1** | **Quintile 2** | **Quintile 3** | **Quintile 4** | **Quintile 5** |
| --- | --- | --- | --- | --- | --- |
| *<40 years* | *200 (3)* | *193 (5)* | *198 (4)* | *193 (2)* | *192 (2)* |
| eCRF (mL∙kg^-1^∙min^-1^) | 39.7 ± 3.6 | 45.4 ± 1.1 | 48.6 ± 0.8 | 51.7 ± 1.0 | 56.9 ± 4.1 |
| *40-49 years* | *317 (23)* | *312 (16)* | *313 (11)* | *318 (19)* | *306 (4)* |
| eCRF (mL∙kg^-1^∙min^-1^) | 35.7 ± 3.1 | 40.8 ± 1.0 | 44.0 ± 0.9 | 47.2 ± 1.0 | 54.3 ± 5.2 |
| *50-59 years* | *379 (43)* | *384 (36)* | *370 (31)* | *368 (42)* | *371 (26)* |
| eCRF (mL∙kg^-1^∙min^-1^) | 32.2 ± 2.9 | 37.0 ± 0.9 | 39.6 ± 0.7 | 42.2 ± 0.8 | 47.5 ± 4.2 |
| *60-69 years* | *338 (46)* | *339 (46)* | *333 (47)* | *336 (33)* | *335 (37)* |
| eCRF (mL∙kg^-1^∙min^-1^) | 29.3 ± 2.5 | 33.9 ± 0.9 | 36.5 ± 0.7 | 39.2 ± 0.8 | 44.5 ± 3.9 |
| *≥70 years* | *64 (18)* | *63 (19)* | *65 (20)* | *62 (20)* | *63 (15)* |
| eCRF (mL∙kg^-1^∙min^-1^) | 26.8 ± 2.4 | 31.2 ± 0.6 | 33.9 ± 0.9 | 36.8 ± 0.7 | 40.7 ± 2.7 |
| *Total, n (mi)* | *1298 (113)* | *1291 (122)* | *1279 (113)* | *1277 (107)* | *1267 (84)* |
| SHR (95%CI) | Ref. | **0.82 (0.64-1.05)** | **0.70 (0.55-0.90)** | **0.67 (0.52-0.86)** | **0.71 (0.54-0.93)** |

Data are adjusted for education, diet quality, smoking, alcohol intake, study survey, and age as timescale. Number of participants (MI) and their mean ± SD of eCRF are shown per 10-year age group, and as merged N (mi). eCRF=estimated cardiorespiratory fitness. SHR=subdistributed hazard ratio, CI=confidence interval, MI=myocardial infarction. SD=standard deviation. Bold number indicate significant association.

# **Supplementary Table S13. The association between estimated cardiorespiratory fitness and myocardial infarction in those with ≥5 years follow-up time. The Tromsø Study 1994-2014.**

|  | **Females** | **Males** |
| --- | --- | --- |
|  | *N (MI)* | *N (MI)* |
|  | *7494 (340)* | *5947 (419)* |
| Arbitrary eCRF values | SHR (95%CI) | SHR (95%CI) |
| 20 mL∙kg^-1^∙min^-1^ | **1.30 (1.10-1.55)** | N/A |
| 25 mL∙kg^-1^∙min^-1^ | *Ref.* | 1.29 (1.07-1.56) |
| 30 mL∙kg^-1^∙min^-1^ | **0.72 (0.62-0.82)** | 1.10 (1.02-1.19) |
| 32 mL∙kg^-1^∙min^-1^ | **0.57 (0.47-0.69)** | *Ref.* |
| 35 mL∙kg^-1^∙min^-1^ | **0.36 (0.25-0.52)** | **0.91 (0.85-0.97)** |
| 40 mL∙kg^-1^∙min^-1^ | **0.13 (0.05-0.33)** | **0.75 (0.63-0.89)** |
| 45 mL∙kg^-1^∙min^-1^ | **0.04 (0.01-0.22)** | **0.63 (0.49-0.80)** |
| 50 mL∙kg^-1^∙min^-1^ | **0.02 (0.001-0.16)** | **0.53 (0.34-0.84)** |
| 55 mL∙kg^-1^∙min^-1^ | N/A | **0.45 (0.22-0.91)** |
| 60 mL∙kg^-1^∙min^-1^ | N/A | **0.38 (0.14-1.01)** |

Data are adjusted for education, diet, smoking, alcohol intake and study survey. Values are arbitrary values of the restricted cubic spline models. eCRF=estimated cardiorespiratory fitness. SHR=subdistributed hazard ratio, CI=confidence interval, MI=myocardial infarction. Bold number indicate significant association.

# **Supplementary Figure S1. Directed acyclic graph for the hypothesized association.**


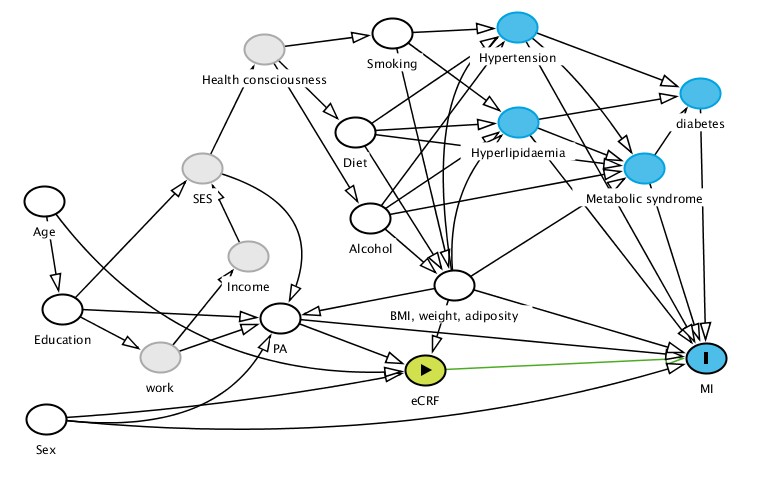


Green circle (with arrow)=exposure, Blue circle (I)=outcome, White circle=adjusted variable, Blue circle= ancestor of outcome, Green line=causal path. PA=physical activity, BMI=body mass index, eCRF=estimated cardiorespiratory fitness, SES=socioeconomic status, MI=myocardial infarction.

# **Supplementary Figure S2.** Log-log survival plot of subdistributed hazards of smoking in females.


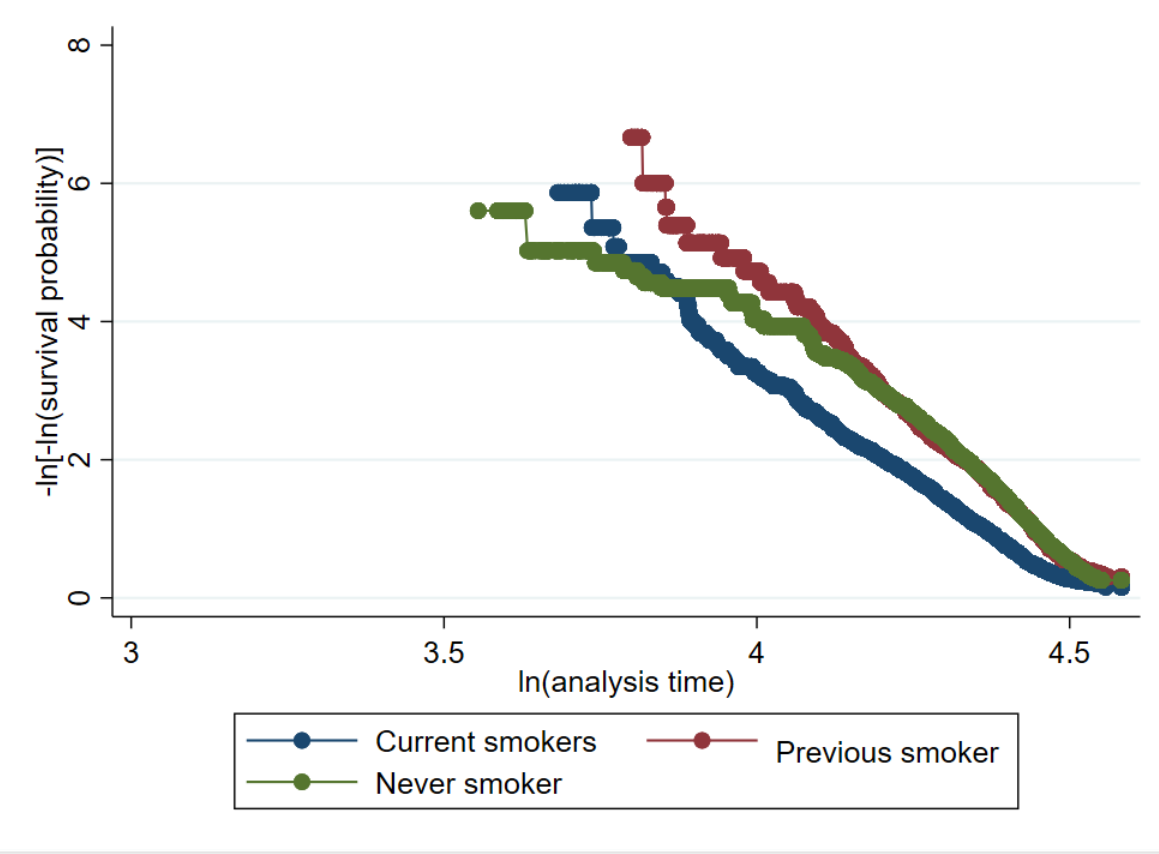


# **Supplementary Figure S3.** Log-log survival plot of subdistributed hazards of smoking in males.


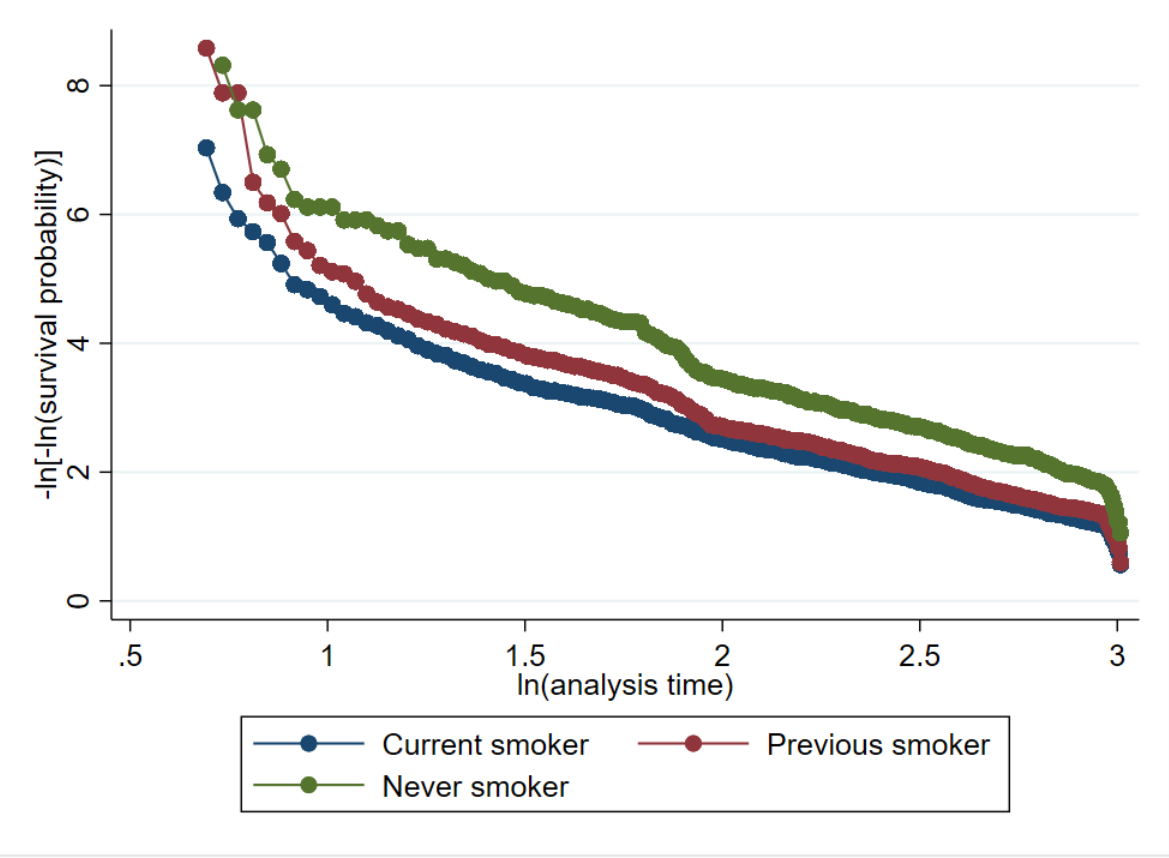


# **Supplementary Figure S4.** Flow chart of included participants


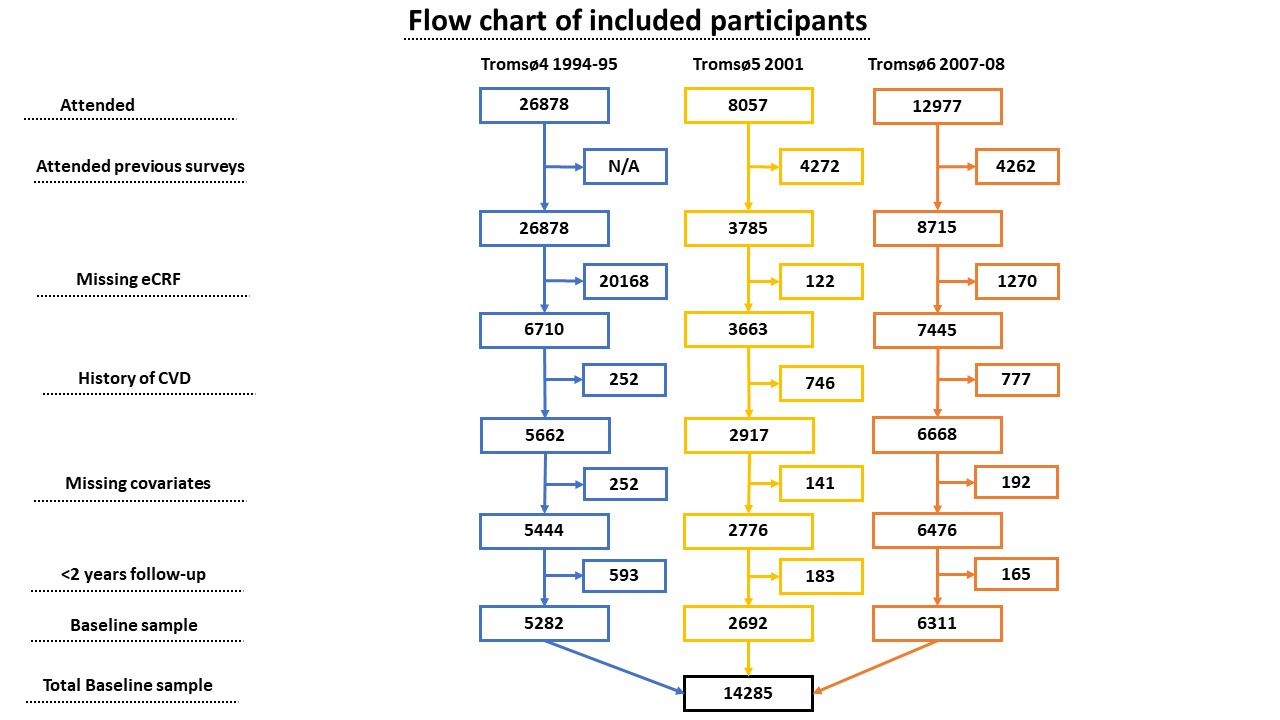


CVD=cardiovascular disease. eCRF=estimated cardiorespiratory fitness.

# **References for the supplementary materials**

1. Nauman J, Nes BM, Lavie CJ, Jackson AS, Sui X, Coombes JS, et al. Prediction of Cardiovascular Mortality by Estimated Cardiorespiratory Fitness Independent of Traditional Risk Factors: The HUNT Study. Mayo Clin Proc. 2017;92(2):218-27.

2. Nes BM, Janszky I, Vatten LJ, Nilsen TI, Aspenes ST, Wisløff U. Estimating V·O 2peak from a nonexercise prediction model: the HUNT Study, Norway. Med Sci Sports Exerc. 2011;43(11):2024-30.

3. Graff-Iversen S, Anderssen SA, Holme IM, Jenum AK, Raastad T. Two short questionnaires on leisure-time physical activity compared with serum lipids, anthropometric measurements and aerobic power in a suburban population from Oslo, Norway. Eur J Epidemiol. 2008;23(3):167-74.

4. Bull FC, Al-Ansari SS, Biddle S, Borodulin K, Buman MP, Cardon G, et al. World Health Organization 2020 guidelines on physical activity and sedentary behaviour. Br J Sport Med. 2020;54(24):1451-62.

5. National recommendations for Nutrition and Physical Activity Oslo: Norwegian Directorate of Health 2014.

6. Ross R, Neeland IJ, Yamashita S, Shai I, Seidell J, Magni P, et al. Waist circumference as a vital sign in clinical practice: a Consensus Statement from the IAS and ICCR Working Group on Visceral Obesity. Nat Rev Endocrinol. 2020;16(3):177-89.

7. Alberti KG, Zimmet P, Shaw J. The metabolic syndrome--a new worldwide definition. Lancet. 2005;366(9491):1059-62.

8. Sagelv EH, Hopstock LA, Johansson J, Hansen BH, Brage S, Horsch A, et al. Criterion validity of two physical activity and one sedentary time questionnaire against accelerometry in a large cohort of adults and older adults. BMJ Open Sport Exerc Med. 2020;6(1):e000661.
